# *Nicotiana benthamiana* is a suitable transient system for high-level expression of an active inhibitor of cotton boll weevil α-amylase

**DOI:** 10.1186/s12896-019-0507-9

**Published:** 2019-03-08

**Authors:** Guilherme Souza Prado, Pingdwende Kader Aziz Bamogo, Joel Antônio Cordeiro de Abreu, François-Xavier Gillet, Vanessa Olinto dos Santos, Maria Cristina Mattar Silva, Jean-Paul Brizard, Marcelo Porto Bemquerer, Martine Bangratz, Christophe Brugidou, Drissa Sérémé, Maria Fatima Grossi-de-Sa, Séverine Lacombe

**Affiliations:** 1 Embrapa Genetic Resources and Biotechnology, Brasília, DF Brazil; 2 Catholic University of Brasília, Brasília, DF Brazil; 3 IRD, CIRAD, Université Montpellier, Interactions Plantes Microorganismes et Environnement (IPME), Montpellier, France; 4 INERA/LMI Patho-Bios, Institut de L’Environnement et de Recherches Agricoles (INERA), Laboratoire de Virologie et de Biotechnologies Végétales, Ouagadougou, Burkina Faso

**Keywords:** Transient protein expression, α-amylase inhibitors, Gene silencing suppressors, Cotton boll weevil

## Abstract

**Background:**

Insect resistance in crops represents a main challenge for agriculture. Transgenic approaches based on proteins displaying insect resistance properties are widely used as efficient breeding strategies. To extend the spectrum of targeted pathogens and overtake the development of resistance, molecular evolution strategies have been used on genes encoding these proteins to generate thousands of variants with new or improved functions. The cotton boll weevil (*Anthonomus grandis*) is one of the major pests of cotton in the Americas. An α-amylase inhibitor (α-AIC3) variant previously developed via molecular evolution strategy showed inhibitory activity against *A. grandis* α-amylase (AGA).

**Results:**

We produced in a few days considerable amounts of α-AIC3 using an optimised transient heterologous expression system in *Nicotiana benthamiana*. This high α-AIC3 accumulation allowed its structural and functional characterizations. We demonstrated via MALDI-TOF MS/MS technique that the protein was processed as expected. It could inhibit up to 100% of AGA biological activity whereas it did not act on α-amylase of two non-pathogenic insects. These data confirmed that *N. benthamiana* is a suitable and simple system for high-level production of biologically active α-AIC3. Based on other benefits such as economic, health and environmental that need to be considerate, our data suggested that α-AIC3 could be a very promising candidate for the production of transgenic crops resistant to cotton boll weevil without lethal effect on at least two non-pathogenic insects.

**Conclusions:**

We propose this expression system can be complementary to molecular evolution strategies to identify the most promising variants before starting long-lasting stable transgenic programs.

## Background

Biotic stresses such as insect pests induce dramatic damages in crops throughout the world, leading to significant losses for growers. To defend against these stresses, chemical treatments are largely used. However, due to health, environmental and cost concerns, for years attention has focused on genetic resistance, both in terms of conventional and transgenic applications [1, 2].

The most common transgenic plants displaying insect resistance (IR) carry genes encoding crystal toxins (Cry) from the soil bacterium *Bacillus thuringiensis* (*Bt*). Cry proteins solubilize in the insect midgut, where they become active and lead to cell lysis and insect death. Cry proteins are toxic to insects but not to humans or other vertebrates [3]. Despite a quite narrow range of control pathogens and low accumulation levels in plants, *Bt* IR crop plants represent one of the most successful achievements in plant transgene technology [2]. Currently, several *Bt* plants, including corn, cotton and soybean, grow under field conditions worldwide [4]. However, lack of high dose *cry* expression in plants still can lead to the selection of insect varieties that acquire resistance against the toxic effects of the Cry molecules via adaptation [5].

On the other hand, plants are equipped with natural defence systems against pests such as insects. These defences mainly involve antimetabolite proteins that induce alterations to the digestive system of insect pests. The transfer of proteinase inhibitor genes from one plant to another has been widely used to develop insect-resistant plants [6–8]. For example, when expressed in *Nicotiana benthamiana*, a beetroot gene encoding a proteinase inhibitor induces resistance to lepidopteran insect pests [9]. Lectins are plant carbohydrate-binding proteins that present a high toxicity to phytophagous insects [10]. Lectins have been used in genetic transformation to provide resistance against spider mite in papaya [11]. Chitinases are also plant-expressed proteins that can provide IR when expressed in a transgenic context [12, 13].

Alpha-amylase inhibitors (α-AI) produced in common bean (*Phaseolus vulgaris*) and other *Phaseolus* species act on α-amylase present in insect guts by inhibiting the processing of complex sugars and, consequently, the growth of insect larvae [14]. They exist as two isoforms, α-AI1 and α-AI2, that undergo proteolytic cleavage from a preprotein to two polypeptides: α- and β-subunits [15]. In addition, amino acid hydrolysis occurs at the C-terminal ends of both α- and β-subunits, giving rise to 10 and 15 kDa chains, respectively [16]. Even if the unprocessed and processed forms accumulated in plants, it has been shown that proteolysis is required for inhibitory activity [15]. Despite a relatively high similarity, α-AI1 and α-AI2 act on specific and distinct spectra of insect α-amylases [14]. Transgenic processes to express bean α-AI have been widely used on several plant species for the improvement of IR [17–20].

Despite the efficiency of these IR strategies, the spectrum of insects controlled by any given protein is quite narrow. Moreover, whatever the controlling strategy is, it must face the development of resistant insects. Hence, to extend the spectrum of target pathogens and to overtake the development of insect resistance, molecular evolution strategies have been used on original IR proteins to generate thousands of variants with potentially new or improved functions [21, 22]. New resistances have been identified from these libraries for the cotton boll weevil (*A. grandis*), sugarcane giant borer (*Telchin licus licus*) and mustard aphid (*Acyrthosiphon pisum*) [23–26]. These findings highlight the importance of the variant libraries to create new IR to harmful insect pests that act on major crops worldwide. However, even with this important agricultural interest, a deep characterization of these proteins is required to demonstrate their economic interest and safety impact such as allergenic issues [27].

Systems allowing low-cost and rapid screening of these libraries are necessary to identify the most promising variants before starting long-term and costly transgenic programmes. Cry and trypsin inhibitor variants are expressed in phage systems before in vitro screening of inhibitory activity [23–25]. However, this phage-based system is not suitable for plant variants requiring post-translational modifications for their activities, such as α-AI. Moreover, the final goal is to express these variants in plants, implying that they would be processed by the plant cell machinery. Consequently, plant-based systems could be more convenient than phage- or prokaryote-based systems to screen these variants and select the most promising ones. The model plant *Arabidopsis thaliana* has been used to stably express α-AI variants. This system allowed the identification of a very promising variant, α-AIC3 that was able to inhibit 77% of the α-amylases from the insect *A. grandis*, whereas the original α-AI forms were ineffective. This variant differs from the original sequence by several amino acid changes induced by the molecular evolution strategy performed [26]. This outcome represents an important finding for the cotton culture in the Americas, where *A. grandis* is among the major insect pests. Consequently a deep characterization of this variant should be done before starting a promising transgenic cotton program. However, *A. thaliana* transgenic-based screenings may not be suitable for evaluating potentially interesting proteins from thousands of variant libraries. Therefore, in order to characterize accurately such protein variants, it is crucial to establish an alternative and robust plant-based expression system that allows the expression of recombinant proteins at high yield and with accuracy in terms of post-translational modifications.

In recent years, advances in biotechnology have led to the emergence of plants as bioreactors for the production of proteins of interest not only in stable transgenic systems but also in transient systems [28]. The first crucial advance was the use of transient expression systems relying on *Agrobacterium* as a vector to deliver DNA encoding proteins of interest directly into leaf cells by syringe infiltration – so-called agroinfiltration [29]. Moreover, protein production can be increased by the co-expression of viral proteins displaying suppression of gene silencing activity. Indeed, the presence of such viral proteins in transient expression systems allows overcoming the gene silencing triggered by the plant defence machinery to specifically degrade foreign nucleic acids. Consequently, the yield of the protein of interest is dramatically increased by 50 fold or more [30, 31].

Here, we describe a high-yielding, easier, quicker and cheaper system compared to the stable transformation of *A. thaliana*. This well-known system is based on the transient expression of the protein of interest in *N. benthamiana* leaves (see for review [32]). As previously described, a combination of three viral suppressors of gene silencing are used to improve the expression in terms of accumulation levels [31]. We focused on an α-AIC3 variant that was previously demonstrated to act on one of the most damaging insects to cotton culture in the Americas – the cotton boll weevil (*A. grandis*) [26]. We showed that these proteins that were transiently expressed in *N. benthamiana* leaves, accumulated at high levels and exhibited their expected post-translation maturation and in vitro function on the target insect enzyme. We proposed this system to be complementary to molecular evolution strategies to allow easy selection and characterization (within a few days) of the most promising variants from molecular evolution libraries before starting stable transgenic programs.

## Results

### α-AIC3 expression in *N. benthamiana* leaves

**Fig. 1:**
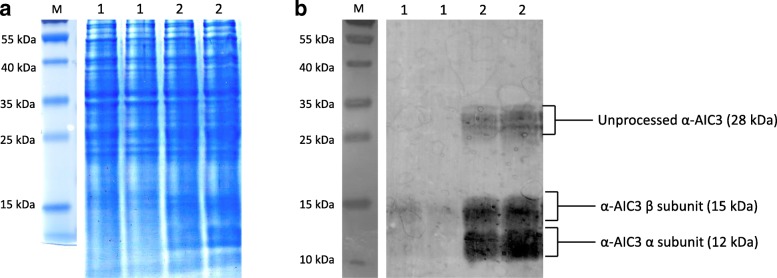
Detection of α-AIC3 expression in presence of gene silencing suppressor combination (P0, P1 and P19). **a**- Coomassie Blue-stained 15% SDS-PAGE consisting of 40 μg of total protein from crude extracts of pBIN61 samples (1) and pBIN61:α-AIC3 samples (2) from *N. benthamiana* leaves co-expressing these vectors with the three gene silencing suppressors . **b**- Western blot of corresponding Coomassie Blue-stained gel using a specific primary anti-α-AIC3 antibody. Expected bands for whole and unprocessed α-AIC3 (27 kDa), as well as for its subunits (α-subunit, 12 kDa, and β-subunit, 15 kDa), are shown. M: Molecular marker

To optimize the accumulation of α-AIC3 in *N. benthamiana* leaves, the *aic3* gene was transiently co-expressed in 4-week-old wild-type *N. benthamiana* plants together with genes encoding three viral gene silencing suppressors. It has been previously demonstrated that these suppressors act synergistically by inhibiting three different steps of the gene silencing defence mechanism [31]. Agroinfiltrated leaf regions were collected at 5 dpi and weighted, after which protein was extracted. A total of 40 μg of soluble proteins representing approximately 10 mg of fresh leaves was separated by 15% (m/v) SDS-PAGE and blotted onto a nitrocellulose membrane. The Coomassie Blue-stained gel (Fig. 1a) showed additional bands of lower molecular mass for samples 2 (pBIN61:α-AIC3) compared to samples 1 (pBIN61), suggesting that this difference was due to the *aic3* gene expression and protein accumulation in the leaves. This result was confirmed by Western blot (Fig. 1b) using a specific anti-α-AIC3 primary antibody; samples 1 did not show any visible band or signal, but samples 2 presented a pattern composed of three intense bands. The lower bands were very intense and referred to the processed α-AIC3 forms, which may correspond to α- and β-subunits of 12 kDa and 15 kDa, respectively. Moreover, bands of higher molecular weight also appeared that were approximately 28 kDa, strongly suggesting that they correspond to the unprocessed forms of α-AIC3; these bands had a less intense signal than the bands attributed to the processed subunits. The results here indicate that α-AIC3 was successfully expressed and mostly processed according to the expected proteolytic processing. Furthermore, the generated bands were not linear but dispersed. These patterns suggest several isoforms that could result from the expected post-translational maturation processes for these inhibitors including amino acid hydrolysis at the C-terminus ends of both subunits and glycosylation [16]. However, despite their accurate size, we cannot exclude that the observed bands were due to protein aggregation or degradation. The following structural and functional characterization were performed to exclude this possibility.

### α-AIC3 yield and expression level analysis and protein purification

**Fig. 2:**
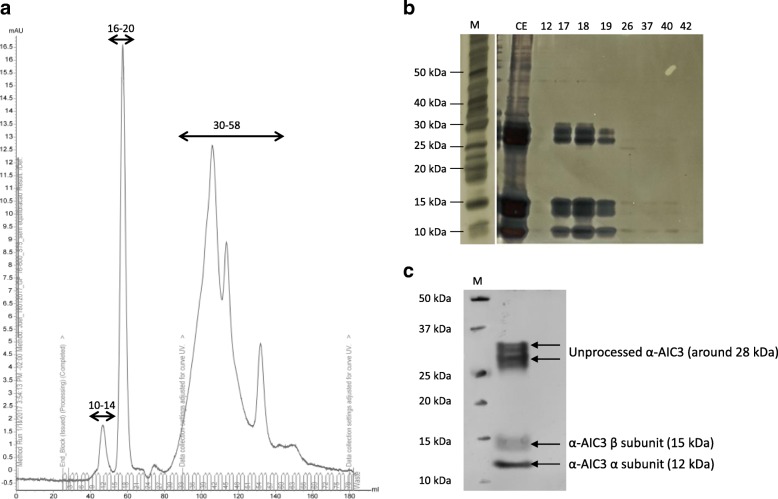
α-AIC3 purification through size exclusion chromatography. **a**- Chromatogram generated from molecular size exclusion chromatography of α-AIC3-expressing *N. benthamiana* extracts after dialysis against water. The indicated peaks comprise fractions 10–14, 16–20 and 30–58. A total of 180 mL of eluted volume was obtained, distributed in 90 fractions of 2 mL each. Software: UNICORN™ 6.4 (GE Healthcare). **b**- Silver-stained 15% SDS-PAGE of selected SEC fractions (15 μL). CE: crude extract; W: washing; numbers: selected SEC fractions. **c-** Western blot of 15% SDS-PAGE gel using a specific primary anti-α-AIC3 antibody. Sample analysed consists of combined fractions 17, 18 and 19 of purified and concentrated α-AIC3. The four bands analysed by mass spectrometry are indicated. M: Molecular marker

The yield of the protein of interest in the dialyzed samples was measured by indirect ELISA using a specific anti-α-AIC3 primary antibody. The expression level of α-AIC3 was estimated for the pBIN61:α-AIC3 samples, considering pBIN61 samples as negative controls. In the first experiment, 13.6 ng of α-AIC3 out of 40 ng of total protein were detected, indicating a yield of 34% of Total Soluble Proteins (TSP) for the heterologous protein. Based on the percentage of the specific expression of α-AIC3, this amount corresponded to a yield of 0.1 mg/g fresh weight (FW) tissue or 100 mg/kg FW. In another experiment, 70.4 ng of α-AIC3 of 160 ng of total protein was detected, indicating a yield of 44% TSP for the heterologous protein and corresponding to 0.15 mg/g FW or 150 mg/kg FW. For the protein purification, a dialyzed extract was used, and proteins were loaded on a gel-filtration column for performing size exclusion chromatography (SEC). A total of 90 fractions consisting of 2 mL each were obtained. The chromatograms showed different peaks for fractions 10–14, 16–20 and 30–58 (Fig. 2a). Hence, some fractions (12, 17, 18, 19, 26, 37, 40, and 42) from each peak were selected to perform electrophoresis and separate samples to further identify presence of α-AIC3 subunits. Silver staining demonstrated that fractions 17, 18 and 19 (Fig. 2b) presented expected bands for α-AIC3. Indeed, these patterns were very similar to the one previously revealed via Western blotting using a specific anti-α-AIC3 primary antibody (Fig. 1b). Based on these results, these fractions were pooled concentrated and separated again for Coomassie Blue staining and western blotting. Four main bands were clearly detected by western blotting at the expected size for unprocessed (two bands around 28 kDa), β (15 kDa) and α subunits (12 kDa) (Fig. 2c). Corresponding bands visualized on Coomassie staining gel were excised to structurally characterize the proteins and confirm identity with the protein of interest.

### Structural characterization

**Fig. 3:**
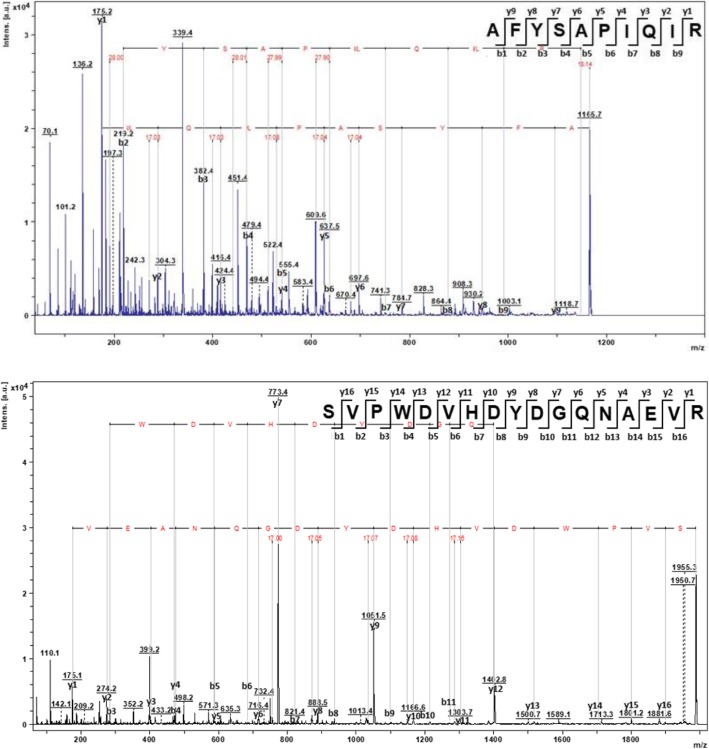
MALDI-TOF MS/MS spectra of fragmented peptides from α-AIC3. Above: parent ion corresponding to an α-subunit peptide [M + H]^+^ = 1165.7 Da; predicted sequence: AFYSAPIQIR. Below: parent ion corresponding to a β-subunit peptide [M + H]^+^ = 1986.7 Da; predicted sequence: SVPWDVHDYDGQNAEVR. Software: FlexAnalysis 3.3 (Bruker Daltonics)

**Fig. 4:**
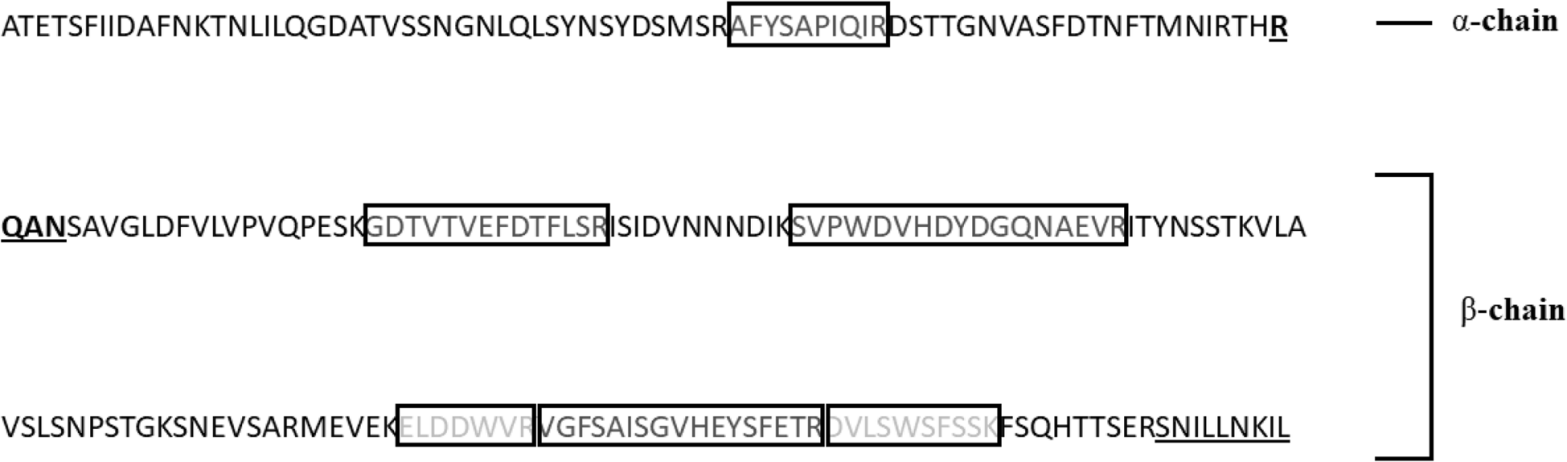
Total sequenced peptides from the α-AIC3 chain. α-AIC3 whole sequence, showing amino acid residues of the α- and β-subunits; the respective peptides were identified, fragmented and sequenced via MALDI-TOF MS/MS and are highlighted inside the rectangles. In total, six peptides were sequenced, one for the α-subunit and five for the β-subunit. C-terminal end peptides, which were cleaved off to yield the mature subunits, for both subunits are underlined in the figure

Spots were excised from the four bands and prepared for MALDI-TOF MS/MS analysis. Spectra of the generated peptides were fragmented, some of which are shown in Fig. 3 with respect to both α-AIC3 subunits. For the α-subunit, one of the four possible tryptic peptides was detected and confirmed after sequencing: AFYSAPIQIR. This finding indicates a coverage of 10 of 73 amino acid residues for the α-subunit, resulting in 14% coverage. For the β-subunit, five of the twelve possible tryptic peptides were detected and confirmed after sequencing: GDTVTVEFDTFLSR, SVPWDVHDYDGQNAEVR, ELDDWVR, VGFSAISGVHEYSFETR and DVLSWSFSSK. This finding indicates a coverage of 65 of 135 residues for the β-subunit, resulting in 48% coverage. In total, six peptides were detected, sequenced and confirmed, indicating a coverage of 75 of 221 residues for α-AIC3 or 34% coverage (Fig. 4). The peptide of the α-subunit was found in all samples corresponding to bands at 28 kDa, 25 kDa and 12 kDa. The five peptides of the β-subunit were found in the samples related to bands 28 kDa, 25 kDa and15 kDa. This finding strongly supports that bands at 15 kDa and 12 kDa represent the β- and α-subunits, respectively, and that bands 28 kDa and 25 kDa represent the whole unprocessed protein, since it contains sequences of both subunits. However, peptides detected do not cover both N- and C-terminus ends of each subunit. Consequently, we cannot exclude that subunits were not intact.

### Functional characterization

**Fig. 5:**
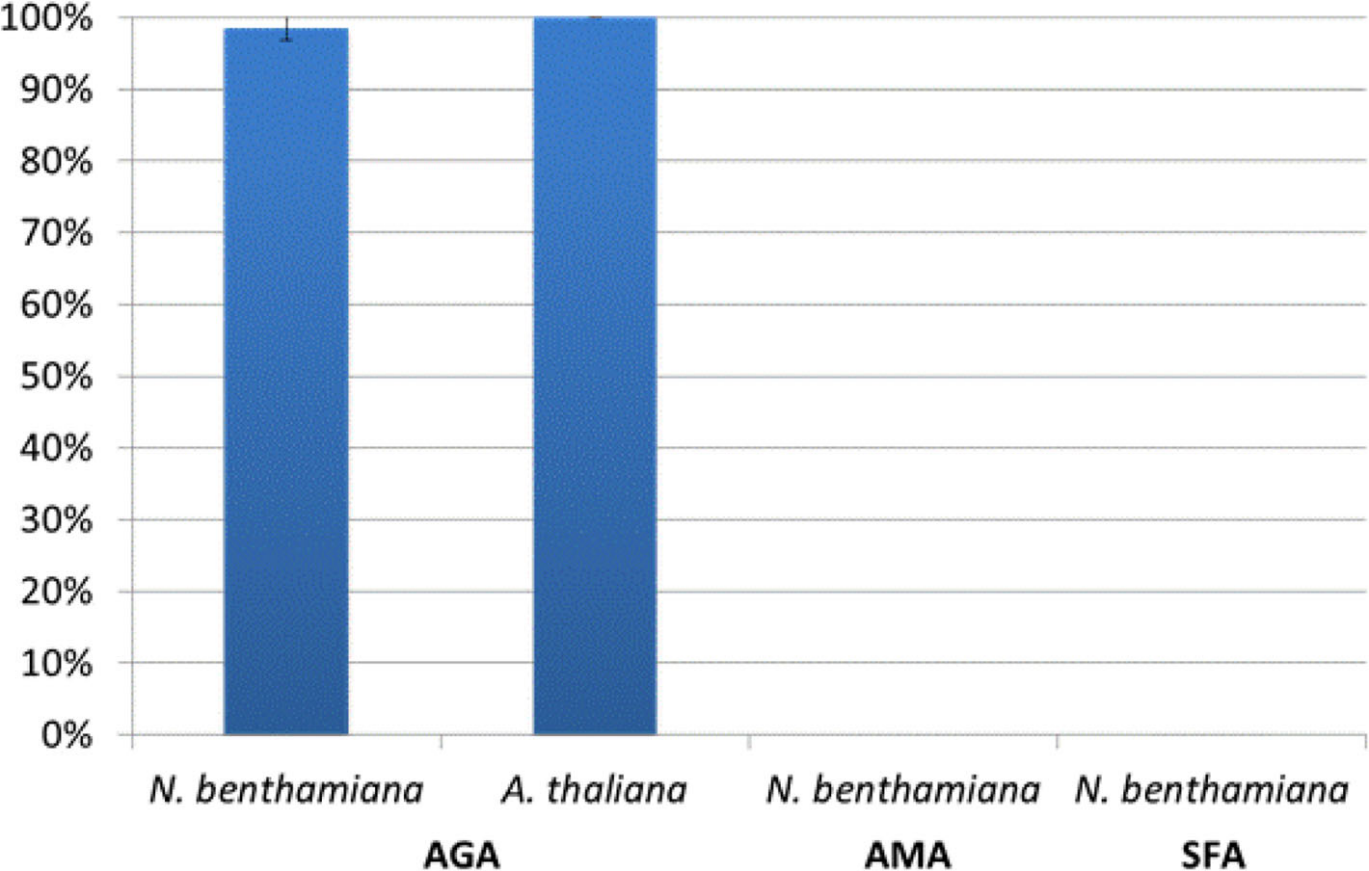
α-AIC3 inhibitory level against target (AGA – *Anthonomus grandis* amylase) and non-target (AMA – *Apis mellifera* – and SFA – *Spodoptera frugiperda*) enzymes. The inhibition levels presented here are based on 100 μg of total soluble protein. The assay results were generated based on three independent experiments. Error bars represent the standard deviation

Based on the DNS method, the α-AIC3-containing samples of *N. benthamiana* showed an average inhibition of 98.5% in *A.grandis* α-amylase (AGA) activity when using 1 unit of enzyme and 100 μg of soluble protein. This inhibition level was validated based on the inhibition obtained using the same amount of *A. thaliana* α-AIC3-containing samples, which completely inhibited the AGA activity. The experiments were repeated for extracts from three different agroinfiltrations. These results showed that the AGA activity inhibition level varied from 96.7 to 100% (Fig. 5). The same extracts of the third agroinfiltration were simultaneously used to assess the inhibition level for AMA and SFA, and did not show any significant inhibition activity (Fig. 5), since absorbances were the same for reactions with or without α-AIC3 and containing active *Apis mellifera* amylase (AMA) and *Spodoptera frugiperda* amylase (SFA) enzymes. Hence, regardless of any assays using AMA and SFA specific inhibitor controls because of their currently unavailability, these data suggested that α-AIC3 produced in *N. benthamiana* was unable to inhibit α-amylase from *A. mellifera* and *S. frugiperda.* Altogether, these results showed that the protein of interest exhibits its expected activity on AGA but exhibits no inhibitory activity against the amylases of these non-target insect species.

## Discussion

The work presented here demonstrates that *N. benthamiana* coupled with the use of a cocktail of gene silencing suppressors is a suitable system for quickly and easily producing a quite high level of an α-AI variant, α-AIC3. The yield was estimated in the range of 100 to 150 mg/kg FW. Plant biosystem yield for proteins of interest is quite variable, reaching up to 2 g/kg of FW in the case of optimised viral based technology [33]. Here, we are in the upper range for non- viral based technology. Moreover, we demonstrated that all the expected α-subunit, β-subunit and unprocessed forms accumulated, mostly consisting of the processed α- and β-subunits. Finally, the expected protein functionality as an inhibitor of AGA was demonstrated, and α-AIC3 did not inhibit the α-amylases of two non-target insects tested (*A. mellifera* and *S. frugiperda*), preliminarily suggesting that α-AIC3 is biologically and environmentally safe. However, it is recommended to perform in vivo studies of α-AIC3, giving rise to an even more realistic result regarding the protein yield and safety,furnishing data concerning the feasibility to produce genetically modified cotton plants that could be resistant to *A. grandis* without triggering biosafety traits such as environmental imbalances or allergenicity.

Concerning the inhibitory assays, we could achieve up to 100% inhibition of AGA using 100 μg of *N. benthamiana* extracts, indicating that up to 44 μg of α-AIC3 were used to inhibit 1 U of AGA, while the complete inhibition of AGA was also achieved using the same amount of total protein from *A. thaliana* seeds. Silva et al. [26] demonstrated that the inhibitory activity was 77% when using 85 μg of total protein from *A. thaliana* leaves expressing this inhibitor at an expression level of 0.2% of TSP, which is very low compared to the level in transient expression obtained here. This means that, from the total protein, approximately 170 ng of α-AIC3 are necessary to inhibit 77% of the AGA activity, and in our study, we can estimate that approximately 200 ng of α-AIC3 are enough to inhibit 1 U of AGA if considering similar expression levels of α-AIC3 in seeds as in leaves for transgenic *Arabidopsis* as reported in the case of 35S transgenic constructs [34]. These reproducible *Arabidopsis* values validate the functional assay used here.

For *N. benthamiana* extracts, the amount of inhibitor used to inhibit completely the AGA activity was much higher, since 100 μg of soluble proteins contain up to 44 μg of α-AIC3 (considering an expression level of 44% TSP). As the kinetic parameters of this enzymatic assay were not known, we cannot exclude that this high amount of inhibitor present in *N. benthamiana* extract saturated the assay. Thus, fewer protein amounts could have triggered similar inhibition levels. Furthermore, we must also consider that the amount of useful α-AIC3, i.e., the amount that effectively participates in the enzyme inhibition was considerably lower than 44 μg. Indeed, based on the western (Fig. 1b), part of total α-AIC3 is composed of unprocessed chains, unable to inhibit the AGA activity as the post-translational processing is imperative for the acquisition of biological activity in α-AI proteins [15]. Moreover, from results from MALDI-TOF MS/MS analysis, we cannot determine the amount of α- and β-subunits produced that are fully active.

Regardless of this, the transient expression system remains a suitable alternative to stable expression systems because former exhibits practicality: it is simple as it does not require complex materials nor techniques and provides considerable amounts of protein without the need for regenerating plants and selecting transformants. Dias et al. [35] also used tobacco-based expression to produce an α-amylase inhibitor (αBIII) of *Secale cereale* in *Nicotiana tabacum* seeds via stable expression. This system also yielded low protein levels (0.1–0.29% TSP) that were similar to those in *A. thaliana* [26] and achieved a maximum inhibition of only 41% of AGA activity when using 250 mg of crude protein extract.

Altogether, these data suggest that this *N. benthamiana* transient expression system may be suitable for the rapid, easy and efficient production of α-AI variants obtained from molecular evolution strategies for preliminary functional screening and biosafety studies. The α-AIC3 variant analysed here was identified from a library that consisted of more than 8000 variants [26]. With such transient system, this library could be efficiently exploited to identify variants with new or improved IR functions against major pests as describe above for the potato gene encoding a disease resistant protein against a virus [36, 37].

*Nicotiana*-based transient expression systems have been widely used to express proteins of interest, such as those for vaccines and biopharmaceuticals [28]. Fewer examples have been described for proteins of agricultural interest. Farnham and Baulcombe [36] produced a variant library using random mutagenesis from a potato gene encoding a disease resistant protein (Rx) against a subset of potato virus X (PVX). Those authors used transient expression in *N. tabacum* to screen 1920 variants. Thirteen of those variants induced a cell-death response in the presence of the PVX coat protein, indicative of disease resistance [36]. The same potato gene encoding Rx resistant protein was also used to generate a library of 1500 mutants that were transiently expressed in *N. benthamiana* together with the gene encoding for the Poplar mosaic virus (PopMV) coat protein. This phenomenon allowed the identification of four variants inducing a cell-death response related to resistance to this new virus [37]. Similar to the results reported here, these studies indicate the interest in this dual variant library/*N. benthamiana* transient expression strategy for its easy, rapid, low-cost ability to identify new or improved disease resistance genes from thousands of variants.

Other proteins are known to be associated with IR and have been used in genetic transformation to bring new IR to important crops worldwide [2]. Molecular evolution libraries consisting of thousands of variants have been developed for Cry proteins and proteinase inhibitors. These variants have been screened using phage-based assays to identify variants with new IR functions not present in the original forms [23, 24]. Because the ultimate goal was to exploit these variants in transgenic plants, the *N. benthamiana* transient expression system presented here would be more accurate for the heterologous expression of the variants with the goal of performing functional tests in order to preview the responses of the transformed plant as a definite host. As such, functional tests must serve as a filtering step, as it is more difficult to regenerate several plants displaying a wide selection of candidate variants for additional sorting of promising proteins and events. Therefore, heterologous expression could save time and material and could reduce the complexity of the process for obtaining transformed and commercially feasible events. For this, it is important to gradually characterize the candidates, as was done in this study. An in vitro stage of characterization is needed to validate the proposed activity against the target. However, it is suitable to proceed with an in vivo and complementary stage of assays in which the insects are grown in the presence of the molecules, checking systemic effects in the insect. Once the biological activity is confirmed following this stepwise study, investigations on genetic transformation will be much more reliable since regenerated plants will display the same in vivo observed activity.

Plant breeding has been recently revolutionized with the advent of genome editing technologies allowing precise modifications in genomic sequences with the so-called genome engineering [38, 39]. Several economically important species, such as cotton, are suitable targets for these technologies [40, 41], especially concerning agronomic traits. These technologies have been successfully used in maize, soybean and rice to induce exact mutations in specific genes, leading to herbicide tolerance [42–44]. Resistance development against biotic stresses can also benefit of these technologies as shown by the development of a genome-edited tomato displaying powdery mildew resistance [45] and an engineered cucumber showing broad virus resistance [46]. Based on that, we can speculate that the dual strategy variant library/*N. benthamiana* transient expression allowing identification of variants of interest could be followed by genome editing technologies to precisely induce modifications in the genome of crops. Results presented here suggest that α-AIC3 would be an ideal candidate to evaluate this hypothesis, as well as producing genome-edited plants displaying new or improved IR through α-AI specific modifications. Moreover, whatever the gene of interest, *N. benthamiana* system presented here could be a useful tool to rapidly and easily identify variants that could be integrated in plant genomes through genome editing strategies.

## Conclusions

In this study, we reported successful transient expression of α-amylase inhibitors using *N. benthamiana*-based system with a recent established combination of gene silencing suppressors. We showed that this system is highly suitable for producing variants of mutant inhibitors, which were expressed not only at a very high yield but also with the correct, albeit incomplete, processing, preserving the expected biological function.

## Methods

### Expression vectors and silencing suppressors

The experiments were performed using *Agrobacterium tumefaciens* C58C1 strain harbouring pBIN61:α-AIC3 expression vector for producing the protein of interest or empty pBIN61 vector for negative control. Based on our previous work demonstrating the positive effect of the simultaneous expression of gene silencing suppressors on the accumulation of candidate protein by blocking the gene silencing defence mechanism [31], these additional gene silencing suppressor vectors were used for the co-expression with pBIN61 vectors. They encoded for P0 from Beet western yellow virus (pBIN61:P0 vector) [47], P1 from Rice yellow mottle virus (pCambia1300:P1Tz3 vector) [48] and P19 from Cymbidium ringspot virus (pBIN61:P19 vector) [49]. Each of them was cloned into expression vectors and transformed in *Agrobacterium tumefaciens* C58C1 strain.

### Gene design, synthesis and cloning

The nucleotide sequence for the gene (*aic3*) encoding the α-AIC3 variant was obtained in silico via reverse translation and codon optimization of the α-AIC3 protein sequence [GenBank:AGB50990.1], as reported by Silva et al. [26]. Codon optimization was performed with Gene Designer 2.0 software [50] based on the codon usage table for *N. benthamiana* species, available at Kazusa Codon Usage Database. The nucleotide sequence for the corresponding native signal peptide (MASSNLLSLALFLVLLTHANS) was also retrieved and codon-optimized. The final insert sequence was flanked by 5′-*Xba*I and 3′-*Bam*HI restriction sites, and a Kozak consensus sequence (GCCACC) was inserted immediately upstream of the start codon. No restriction sites for *Xba*I and *Bam*HI were detected within the CDS. SignalP 4.1 Server was used for signal peptide detection and validation. The sequence was synthesized de novo by Epoch Life Science® and cloned into the *Xba*I-*Bam*HI cloning sites of the pUC18 vector to generate pUC18:α-AIC3. The *aic3* gene was then excised from pUC18:α-AIC3 and cloned into the *Xba*I and *Bam*HI sites of the pBIN61 binary expression vector, which was previously described by Bendahmane et al. [51] under the control of the constitutive CaMV 35S promoter and terminator to generate pBIN61:α-AIC3 that was used to transform *A. tumefaciens* strain C58C1 via electroporation. The cloning into the pBIN61 vector was confirmed by sequencing using the M13 forward primer and carried out by Beckman Coulter Genomics®. Cells were also transformed with empty vectors; these cells served as negative controls.

### Agroinfiltration, plant material and experimental conditions

Strains harbouring empty pBIN61, pBIN61:α-AIC3, pBIN61:P0, pCambia1300:P1Tz3 and pBIN61:P19 vectors were separately grown overnight from precultures at 28 °C and 200 rpm in an orbital shaker using LB medium containing rifampicin (100 μg/mL) and kanamycin (50 μg/mL). The cultures were pelleted by centrifugation for 10 min at 4000 *g*, after which the pellets were resuspended in 10 mM MgCl_2_ to a final OD600 of 0.5. Acetosyringone (4-hydroxy-3,5-dimethoxyacetophenone) was added to each suspension to a final concentration of 100 μM for virulence induction, and the suspensions were incubated at 24 °C for 3 h. Agroinfiltration cocktails were prepared by combining cultures for co-infiltration: for the negative control, the pBIN61 culture was combined with the cultures of silencing suppressors (pBIN61:P0:P1:P19, 3:1:1:1, *v*/v:v/v), and the same procedure was employed for protein expression, in which the pBIN61:α-AIC3 culture was combined with the cultures of silencing suppressors (pBIN61-α-AIC3:P0:P1:P19, 3:1:1:1, v/v/v/v). Cocktails were infiltrated into the leaves of 4 weeks old wild-type *N. benthamiana* plants using syringes without needle. Four plants were used for the negative control per experiment, while twelve plants were used for α-AIC3 expression. The plants were placed in a growth chamber and cultivated for 5 days before harvesting (12 h of light per day, 24 °C, 60% relative humidity). Three independent experiments were performed to generate three biological replicates for subsequent molecular analysis.

### Protein extraction, dialysis and concentration

Infiltrated leaf tissues were harvested from the plants at 5 days post-infiltration (dpi). The fresh leaves were combined in their respective groups (negative control and α-AIC3 expression), weighted, frozen in liquid nitrogen and then ground using a mortar and pestle. Protein extraction was performed by adding 700 μL of extraction buffer (20 mM Tris-Cl, 100 mM NaCl, 10 mM Na_2_EDTA·2H_2_O, 25 mM D-glucose, 0.1% Triton X-100, 5 mM EGTA, 5% (v/v) glycerol, 5 mM dithiotreitol, and 1 mM phenylmethanesulfonyl fluoride, pH 7.4) to 300 mg of tissue powder. Crude extracts were incubated on ice for 20 min, strongly shaken for 20 min at 4 °C using a vortex and centrifuged at 14000 *g* for 30 min at 4 °C. The total soluble proteins (TSP) were recovered from the supernatants and dialyzed against water (1 mL of extract per 200 mL of distilled water) using Slide-A-Lyzer™ G2 Dialysis Cassettes (ThermoFisher Scientific) that had a 10 kDa molecular weight cut-off (MWCO). The dialyzed samples were clarified by centrifugation at 14000 *g* for 10 min and quantified by a Bio-Rad® Bradford protein assay [52] based on a bovine serum albumin (BSA) (Sigma Aldrich) standard curve.

### SDS-PAGE and Western blot

A total of 40 μg of protein for each extract was subjected to low-pressure drying, resuspended in 15 μL of pure water and then diluted in protein loading buffer [53] with 2-mercaptoethanol. The samples were incubated at 95 °C for 5 min, loaded and then separated by 15% (m/v) SDS-PAGE. A mirror gel was also made for protein detection via immunoblotting. Proteins were stained with Coomassie Brilliant Blue G-250 or blotted onto a nitrocellulose membrane at 5 V for 20 min in a Trans-Blot® SD semi-dry system (Bio-Rad) after the membrane and gel were treated with blotting buffer (20 mM Tris base, 150 mM glycine, 20% methanol, pH 8.3) for 10 min. Western blot analysis proceeded by blocking the membrane with a 3% (m/v) solution of skimmed milk powder in TBS-T buffer (20 mM Tris base, 150 mM NaCl, 0.1% Tween 20, pH 7.5) for 2 h under shaking. The protein was probed by adding a primary specific anti-α-AIC3 rabbit IgG (GenScript) to the TBS-T buffer (1:2500 of antibody:buffer, or at 0.4 μg.mL^− 1^), after which the membrane was incubated for 2 h under shaking. After six five-minute rinses with TBS-T buffer, the bound antibodies were probed by adding an AP-conjugated secondary goat anti-rabbit IgG (Sigma Aldrich) to the TBS-T buffer (13,000 of antibody:buffer, or 0.3 μg.mL^− 1^), after which the membrane was incubated again for 1 h under shaking. Subsequent washing followed as described, and the proteins were detected using a colorimetric AP substrate reagent kit (Bio-Rad) according to the manufacturer’s instructions.

### ELISA: α-AIC3 quantification

Dialyzed samples were used to estimate the expression level of α-AIC3 in the total protein by indirect ELISAs. Assays were performed in triplicate by coating 96-well microplates with 40 ng or 160 ng of total protein. A standard curve of protein amount (R^2^ = 0.9948) was constructed based on a gradient from 0.2 ng to 200 ng, in a total of 11 dilutions, of both the bacterial and purified β-subunits of α-AIC3 previously produced and kindly provided by Dr. Leonardo Macedo (Embrapa Genetic Resources and Biotechnology, Brasília, Brazil). The samples were diluted in coating buffer (50 mM sodium bicarbonate/carbonate, pH 9.6), and the coated plates were incubated at 4 °C for 18 h. Samples were incubated at 37 °C for 1 h and washed thrice with 200 μL of PBS-T buffer (136 mM NaCl, 3 mM KCl, 10 mM Na_2_HPO_4_, 2 mM KH_2_PO_4_, and 0.05% Tween 20, pH 7.4). The membrane was blocked by using 3% (m/v) gelatin in PBS-T buffer for 2 h at 37 °C. The samples were discarded, washed and incubated together with 100 μL of a primary anti-α-AIC3 antibody (GenScript) diluted in PBS-T buffer with 1% gelatin (1:1000 of antibody:buffer, *v*/v, or at 1 μg.mL^− 1^) for 2.5 h, at 37 °C. The samples were then washed and incubated together with 100 μL of an HRP-conjugated secondary goat anti-rabbit IgG H + L (Bio-Rad) in PBS-T buffer with 1% (m/v) gelatin (1:3000 antibody:buffer, v/v, or at 0.3 μg.mL^− 1^) for 1 h at 37 °C. The samples were detected with 100 μL of a revealing solution as peroxidase substrate consisting of 10 mL of phosphate-citrate buffer (24.3 mM citric acid, 51.4 mM Na_2_HPO_4_, and 0.06% H_2_O_2_, pH 5.0) and 1 mg of 3,3′,5,5′-tetramethylbenzidine (TMB) (Sigma Aldrich). The colour reaction was stopped after 15 min at room temperature with 100 μL of stop solution (3 M H_2_SO_4_). The absorbance values were read at 450 nm using a SpectraMax 190 microplate reader (Molecular Devices), and the samples were analysed according to the appropriate calculations using Excel 2007 software (Microsoft).

### Protein purification

Dialyzed samples of the expressed α-amylase inhibitor were also used for purification via size exclusion chromatography (SEC) using a HiLoad 16/600 Superdex 75 pg (GE Healthcare) 120 mL column. As such, 15 mL of extract was completely dried under reduced pressure and resuspended in 1 mL of equilibration buffer (PBS 1X, 1 mM EDTA, 1 mM EGTA, and 1 mM dithiotreitol, pH 7.4). Afterward, the column was washed with 120 mL of distilled water at a flow rate of 1 min/mL and then equilibrated with 240 mL of equilibration buffer at the same flow rate. The protein solution was loaded on the column, and 180 mL of equilibration buffer was injected at a continuous flow rate of 1 min/mL for elution; fractions were collected every 2 min. Chromatography was performed using an ÄKTAprime plus protein purification system (GE Healthcare), and chromatogram peaks at 280 nm were generated and analysed by UNICORN 6.4 software (GE Healthcare). Ninety fractions (2 mL each) were collected, and 15 μL of each fraction of the different peaks were separated by 15% (m/v) SDS-PAGE for silver staining according to the methods of Switzer et al. [54]. Fractions corresponding to the α-AIC3 peak were combined, lyophilized, resuspended in ultrapure water and quantified. Aliquots of 20 μg of proteins were separated by electrophoresis using 15% (m/v) SDS-PAGE.

### In-gel digestion and mass spectrometry (MALDI-TOF) analysis

Spots of bands were excised from purified α-AIC3 corresponding bands, i.e., processed and unprocessed forms, and prepared for trypsin-based in-gel digestion. The samples were destained three times with 30% (*v*/v) ethanol under vigorous shaking for 20 min. Afterward, samples were dehydrated with a solution of 50% (v/v) acetonitrile (ACN) and 25 mM NH_4_HCO_3_ for 15 min, after which 200 μL of 100% (v/v) ACN was added to the recovered gel pieces, which were then shook for 10 min. The supernatant was discarded, the pieces were dried at room temperature and 15 μL of activated trypsin (Promega), which was prepared in digestion buffer according to the manufacturer’s instructions, was added. The mixture was then incubated on ice for 30 min. Digestion proceeded by adding 25 μL of 50 mM NH_4_HCO_3_ to the samples, which were then incubated at 37 °C for 18 h. The hydrolysis products were collected, desalted, concentrated and purified using C18 resin ZipTip® pipette tips (Merck Millipore) according to the manufacturer’s instructions, although peptides were eluted with 80% (v/v) aqueous ACN. The resulting peptides were dried under reduced pressure and resuspended in 10 μL of ultrapure and sterile water. Molecular mass analyses of α-AIC3 and its fragments were performed by MALDI-TOF MS/MS. A saturated α-cyano-4-hydroxycinnamic acid (CHCA, Sigma Aldrich) solution at 10 mg/mL was prepared in a 1:1 (v/v) aqueous acetonitrile solution containing 0.3% TFA. The solution of the hydrolysis products was mixed with CHCA solution (CHCA:sample, 3:1, v/v), spotted onto a MALDI target plate, and completely dried for crystallization at room temperature before analysis. Desorption/ionization, analysis and detection of peptides were performed using an Autoflex™ Speed mass spectrometer (Bruker Daltonics), and ionization was carried out in positive reflection mode. Spectra were acquired based on external calibration using Protein Calibration Standard II (Bruker Daltonics) in accordance with the manufacturer’s instructions. Peptide fragmentations were performed by using the LIFT™ method [55]. MS/MS spectra were manually interpreted, and the corresponding peptides were sequenced from the b/y series using FlexAnalysis 3.3 software (Bruker Daltonics). The peptide sequences were compared to the data from expected tryptic peptides generated by the theoretical tryptic digestion of α-AIC3 in ExPASy PeptideMass for confirming the already-known sequence and performing coverage analysis.

### In vitro inhibitory assays

#### Activity validation of transiently expressed α-AIC3

The inhibitory activity of *N. benthamiana*-expressed α-AIC3 was first assessed and validated against cotton boll weevil amylase (AGA) based on the comparative inhibitory activity of α-AIC3 previously expressed in *A. thaliana* [26]. The colorimetric assay was performed by measuring the AGA activity using the 3,5-dinitrosalicylic acid (DNS) method adapted from Bernfeld [56] and using 2% (m/v) starch as substrate. Gut extracts as source of α-amylase were prepared by isolating gut from adults of *A. grandis* using a steel blade and mixing with AGA buffer (150 mM succinic acid, 20 mM CaCl_2_, 60 mM NaCl, and 1 mM PMSF, pH 5.0) to a concentration of 0.5 g/mL. The assays were performed with a volume of gut extract containing one unit of α-amylase, which was defined as the amount of enzyme necessary to increase the absorbance (OD550) within 20 min to an amount between 0.11 and 0.15. Seed protein extracts from transgenic and non-transgenic *A. thaliana* were used as a control for α-AIC3 activity, whose transgenic one expressed α-AIC3 at a level of around 0.2% TSP [26]. These extracts were prepared by grinding seeds using a mortar and pestle, mixing each mg of powder with 7 μL of PBS-T buffer (10 mM sodium phosphate, 0. 15 M NaCl, 0.05% (*v*/v) Tween-20, pH 7.5). Crude seed extracts were incubated on ice for 20 min, strongly shaken for 20 min at 4 °C using a vortex and centrifuged at 14000 *g* for 30 min at 4 °C. TSP were recovered from the supernatants and used for performing assays. Negative controls of digestion for all the samples were applied by inactivating the enzyme at 95 °C for five minutes before adding starch to the reaction system. Negative controls were used to prove that the enzyme was heat-inactivated and, thus, to give a background of inhibition to be used in calculations for inhibition level in digestion systems without heat-inactivation. *A. thaliana* seed extract controls were used to validate parameters of the assay based on published data. This validation step allows conclusions concerning *N. benthamiana* extracts, such as the inhibition ability of AGA for the prepared extracts, and the comparison of inhibition level for each α-AIC3 against AGA. All of the reaction systems, i.e., digestions and negative controls, were performed in three technical replicates. We used 100 μg of dried protein resuspended in 75 μL of AGA buffer containing 1 unit of AGA as a source of plant material for each reaction. The absorbance values were recorded at 550 nm using a SpectraMax 190 microplate reader (Molecular Devices), and the samples were analysed using Excel 2007 software (Microsoft). Calculations were based on discounting the absorbance values for respective negative controls of digestion in each sample. Resulting values were used as following: absorbance values for samples containing α-AIC3 were discounted from the values for samples without α-AIC3, and the mean of triplicates indicated the level of activity remaining in each system.

#### Biosafety analysis: Non-target species enzymes

Once the inhibitory activity of the *N. benthamiana* extracts containing α-AIC3 was confirmed against AGA, these samples were used for assaying the inhibitory activity against enzymes of non-target species (*Apis mellifera* amylase – AMA – and *Spodoptera frugiperda* amylase – SFA). Samples at concentrations of 0.5 g/mL of ground whole insects were prepared using either AMA buffer (150 mM succinic acid, 20 mM CaCl_2_, 60 mM NaCl, and 1 mM PMSF, pH 6.5) or SFA buffer (500 mM Tris-Cl, 20 mM CaCl_2_, 60 mM NaCl, and 1 mM PMSF, pH 9.0) based on the recommended values of pH for enzyme activity according to the literature [57, 58]. The assays were performed following the same steps as those of the AGA test, as well as 100 μg of protein from the dialyzed *N. benthamiana* extracts was used. Since there are no specific amylase inhibitors developed, set and available for both insect species, comparison values relative to the absence of activity for AMA and SFA were exclusively derived from heat-inactivated enzyme systems, similarly to the negative control for AGA. The colour reactions were read at 550 nm, after which the appropriate calculations were used to analyse samples and inhibitory activities.

## Abbreviations

ACN: Acetonitrile
AGA: *Anthonomus grandis* α-amylase
AMA: *Apis millefera* amylase
BSA: Bovine serum albumin
*Bt*: *Bacillus thuringiensis*
Cry: Crystal toxin
DNS: 3,5-dinitrosalicylic acid
FW: Fresh weight
IR: Insect resistance
MWCO: Molecular weight cut-off
PopMV: Poplar mosaic virus
PVX: Potato virus
SFA: *Spodoptera frugiperda* amylase
TSP: Total soluble proteins
X SEC: Size exclusion chromatography
α-AI: α-amylase inhibitor
